# Novel GMO-Based Vaccines against Tuberculosis: State of the Art and Biosafety Considerations

**DOI:** 10.3390/vaccines2020463

**Published:** 2014-06-16

**Authors:** Amaya Leunda, Aline Baldo, Martine Goossens, Kris Huygen, Philippe Herman, Marta Romano

**Affiliations:** 1 Biosafety and Biotechnology Unit, Scientific Institute of Public Health, 14 Juliette Wytsman Street, Brussels 1050, Belgium; E-Mails: Aline.Baldo@wiv-isp.be (A.B.); Martine.Goossens@wiv-isp.be (M.G.); Philippe.Herman@wiv-isp.be (P.H.); 2 Immunology Unit, Scientific Institute of Public Health, 642 Engeland Street, Brussels 1180, Belgium; E-Mail: Kris.Huygen@wiv-isp.be

**Keywords:** tuberculosis, vaccine candidate, clinical trial, genetically modified organism, risk assessment, biosafety

## Abstract

Novel efficient vaccines are needed to control tuberculosis (TB), a major cause of morbidity and mortality worldwide. Several TB vaccine candidates are currently in clinical and preclinical development. They fall into two categories, the one of candidates designed as a replacement of the Bacille Calmette Guérin (BCG) to be administered to infants and the one of sub-unit vaccines designed as booster vaccines. The latter are designed as vaccines that will be administered to individuals already vaccinated with BCG (or in the future with a BCG replacement vaccine). In this review we provide up to date information on novel tuberculosis (TB) vaccines in development focusing on the risk assessment of candidates composed of genetically modified organisms (GMO) which are currently evaluated in clinical trials. Indeed, these vaccines administered to volunteers raise biosafety concerns with respect to human health and the environment that need to be assessed and managed.

## 1. Introduction

Tuberculosis (TB) is a contagious infectious disease caused by *Mycobacterium tuberculosis* (*Mtb*) and other mycobacteria belonging to the *Mtb* complex (*M*. *africanum*, *M*. *bovis*, *M*. *canettii*). TB is spread by aerosols of species of the *Mtb* complex, which are shed through cough from open cavitary pulmonary TB patients before onset of multi-drug therapy. It is a major source of morbidity and mortality which has afflicted mankind since its origin [1]. *Mtb* is a pathogenic micro-organism belonging to the risk group (RG) 3 namely a biological agent that can cause severe human disease and may present a risk of spreading to the community [2]. An effective prophylaxis or treatment for infectious diseases caused by agents of RG 3 is usually available. An effective treatment exists against TB except for patients with extremely multi-drug resistant strains (XDR-TB) which are defined as strains resistant to most of the available antibiotics. The airborne route of transmission of *Mtb* and its low infectious dose in humans (ID_50_ 1–10 bacilli) greatly contributes to the final classification of this pathogen into RG 3 [3].

World Health Organization (WHO) reported that in 2012, 8.6 million new cases of TB were notified and that 1.3 million people died from TB [4]. Among the 8.6 million cases of incident TB, 13% were in HIV co-infected individuals, 450,000 were caused by a multi-drug resistant strain (MDR-TB) defined as resistant to both isoniazid and rifampicin, 2.9 million were women and 0.53 million were children. Among the 1.3 million people that died of TB, 320,000 individuals were HIV-positive, 170,000 died of MDR-TB, 410,000 were women and 74,000 were HIV-negative children.

According to the WHO, the TB mortality rate has fallen globally by 45% since 1990 and number of deaths is falling in most parts of the world. This can be attributed to a better access to treatment around the world (via the implementation of treatment programs such as the one of directly observed treatment short-course or DOTS) as well as to the recent development of more rapid diagnostic methods (for example the GeneExpert). Nevertheless, in order to achieve an effective control of TB, novel antibiotics, new treatment schemes and effective vaccines able to prevent the development of the contagious pulmonary form of TB are needed.

Until now, there is no effective vaccine to prevent TB in adults [4]. Bacille Calmette-Guérin (BCG) is a live-attenuated vaccine derived from *M*. *bovis* isolated in 1921 that is still the only licensed vaccine available against TB. Vaccination with BCG was progressively implemented worldwide since the middle of the 20th century and currently, in TB endemic countries, BCG is part of the vaccines administered in the context of the WHO Expanded Program on Immunization (EPI). In non-endemic countries, BCG is recommended only to individuals at increased risk of exposure to *Mtb*. BCG vaccination protects children against TB meningitis and against disseminated disease, but has been found to be of variable efficacy against pulmonary TB (ranging from 0% to 80%) in a number of clinical trials [5,6,7]. Therefore, BCG has a minor impact on transmission of *Mtb* infection.

A major problem in the quest for more efficient TB vaccines is the poor understanding of the immunity that is needed for effective protection. Nevertheless, it is well established that a key role in protection against this intracellular pathogen is played by the cellular arm of the adaptive immune system, particularly by CD4^+^ Th1 (type T helper) cells producing IFN-γ, TNF-α and IL-2 [8]. This is underscored by the clinical association between HIV and TB, by the genetic susceptibility to TB and opportunistic mycobacterial disease of individuals bearing mutations in the IL-12/IL-23–IFN-γ pathway and by the increased risk to develop reactivation TB of individuals treated with anti-TNF-α agents used for a range of inflammatory/autoimmune diseases, such as rheumatoid arthritis and Crohn’s disease [9,10,11]. MHC class I restricted CD8^+^ T cells play also a role in the immune response against *Mtb* through production of cytokines and their lytic activity targeting infected cells. This has been demonstrated by a number of studies in pre-clinical models and with samples isolated from humans [8,12]. The importance of CD8^+^ T cells in the control of latent TB infection and immune protection against reactivation TB in humans was convincingly demonstrated by Bruns *et al* [13]. who have reported that anti-TNF-α immunotherapy with infliximab reduced CD8^+^ T cell-mediated antimicrobial activity against *Mtb* in humans through the interaction of the antibody with cell surface expressed TNF-α on CD8^+^ T cells and their subsequent complement-mediated lysis [13]. Hence, when developing novel TB vaccines, the type of immunity that is mainly sought is cellular adaptive immunity with CD4^+^ Th1 cells and CD8^+^ T cells.

In this review, TB vaccine candidates that are currently evaluated in clinical trials are presented. A particular emphasis is given to the risk assessment of vaccines composed of genetically modified organisms (GMO) such as recombinant BCG and *Mtb* strains or viral-vectored sub-unit vaccines. Indeed, vaccines based on GMO that are administered to volunteers raise biosafety concerns with respect to human health and the environment that need to be assessed and managed [14,15].

## 2. Risk Assessment of Activities Involving GMO-Based Vaccines against TB

### 2.1. General Regulatory Considerations in Europe

Depending on the purpose and the type of activity in the European Union (EU), the use of genetically modified and/or pathogenic (micro-)organisms may fall within the scope of several regulatory provisions (Table 1). As the manipulation of pathogenic micro-organisms may pose a risk related to the exposure of the worker to biological agents, this type of activity is covered by the European Directive 2000/54/EC [2]. Activities involving manipulations of Genetically Modified Micro-organisms (GMM) in a facility may increase the probability of exposure of the population and the environment to these organisms. In this case, a risk assessment should be made in accordance with the provisions of the Directive 2009/41/EC related to the contained use of GMMs [15]. In addition if these activities involve the deliberate release of GMOs (including GMMs) into the environment they should undergo an environmental risk assessment (ERA) according the principles defined in annex II of the European Directive 2001/18/EC [14]. Moreover, activities aiming at commercializing of GMO biopharmaceuticals are covered by the Regulation (EC) No 726/2004 laying down procedures for the authorization and supervision of medicinal products for human and veterinary use [16].

### 2.2. Activities Involving Manipulation of GMO-Based Vaccine Candidates against TB

Development of vaccines involves two main steps: the pre-clinical and the clinical development stage starting when the vaccine is first tested in humans.

**Table 1:** EU’s regulatory framework governing the conduct of clinical trials using genetically modified organisms (GMOs) and/or pathogens and the marketing of medicinal substances containing or consisting of GMOs (adapted from Verheust *et al*. 2012 [17])

Legislation	Main elements	Ref.
Directive 2000/54/EC	This Directive aims at the protection of workers against risks to their health and safety, including the prevention of such risks, arising or likely to arise from exposure to biological agents at work. It requests Member States to determine the nature, degree and duration of worker’s exposure during an activity likely to involve a risk of exposure to biological agents and on the basis of this assessment, to implement adequate protective measures. Biological agents are classified in four risk groups, from the one that is unlikely to cause human disease to a biological agent causing severe disease for which no effective prophylaxis or treatment is available.	[2]
Directive 2001/18/EC	This Directive defines the procedure for granting consent for the deliberate release in the environment and placing on the market of GMOs as or in products. It provides for a common methodology to assess on a case-by-case basis the risks for human health and the environment associated with the release of GMOs. It also introduces compulsory monitoring after GMOs have been placed on the market, as well as compulsory public consultation and GMO labeling.	[14]
Directive 2009/41/EC	This Directive focuses on the contained use of genetically modified micro-organisms (GMMs), *i.e*., any activity involving GMMs for which specific containment measures are used to limit their contact with, and to provide a high level of safety for, the general population and the environment. The Directive requests Member States to assess on a case-by-case basis the risks contained uses may pose and to implement appropriate containment and other protective measures to avoid adverse effects on human health and the environment. Contained uses are classified in four classes, from no or negligible risk to activities of high risk.	[15]
Regulation (EC) No. 726/2004	This Regulation lays down procedures for the authorization, supervision and pharmacovigilance of medicinal products for human and veterinary use. For medicinal products derived from biotechnology, it foresees a compulsory centralized authorization procedure in which the European Medicines Agency is responsible for drawing up opinions on any matter concerning the evaluation of the products.	[16]
Directive 2001/20/EC	This Directive sets out common rules for the authorization and regulatory follow-up of a clinical trial. It aims at protecting human subjects involved in clinical trials and ensuring that the results are credible, by establishing quality, safety and ethical criteria. Approval of trials is the responsibility of individual EU Member States, who are required to evaluate the products used in clinical studies.	[18]

**Figure 1:**
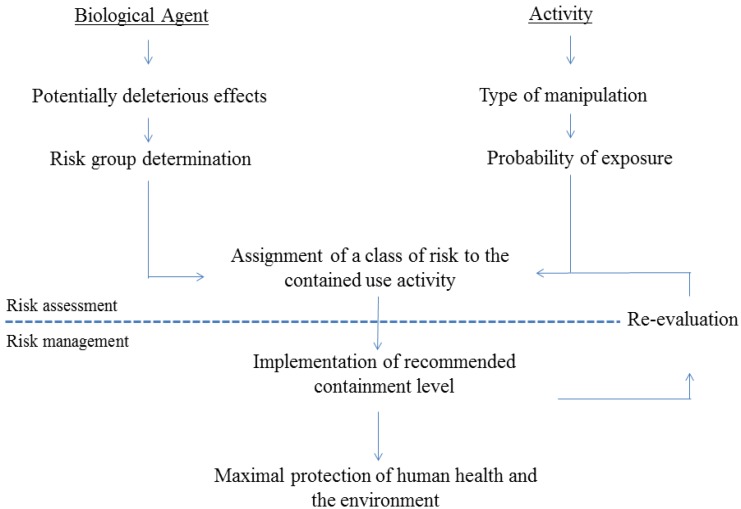
The risk assessment of a “contained use” activity. The risk assessment takes into consideration on one hand, the identification of biological hazards of the genetically modified or pathogenic organism and the determination of its risk group and on the other hand, the nature of the manipulations determining the probability of exposure to potential biological hazards. The risk assessment allows assigning a class of risk to the contained use activity and the implementation of the recommended containment level in order to protect public health and the environment.

During the pre-clinical stages of research and development of novel TB vaccines, a number of studies are performed involving handling pathogenic *Mtb* or the GMO-based vaccine candidate. These research activities are carried out in contained facilities with a set of containment measures in order to protect human health and the environment against an accidental exposure to *Mtb* or the GMO vaccine candidate. A case-by-case risk assessment of these activities is performed to estimate the probability of occurrence and the severity of adverse effects in order to adapt the containment measures (Figure 1). It should identify potentially harmful properties of the biological agent (pathogenic organism genetically modified or not) that is manipulated. The following properties have to be documented to conduct a proper risk assessment: the pathogenicity of the organism, the transmission mode, its infectious dose, its persistence and stability in the environment and the availability of effective prophylaxis or therapy against the disease. Concerning the risk assessment of a GMO, each element used towards the achievement of the genetic modification should be assessed: the recipient organism, the genetic material inserted, the vector and the donor organism. The final organism (genetically modified or not) is then classified into one of the four risk groups according to their relative hazards, RG 1 referring to micro-organisms that are proven non-pathogenic or are unlikely to cause disease (in healthy individuals) and RG 4 referring to micro-organisms highly transmissible that cause a fatal disease for which no treatment or prophylaxis is available [2]. The risk assessment takes also into account the characteristics of the operation performed with the organism that could influence the probability of exposure of workers, the general population and the environment: the scale of the operations (a production activity involving high volumes), the concentration of the organism and the type of manipulations involving the organism (creating infectious aerosols for example).

The case-by-case risk assessment of the activity allows the determination of an adequate containment level (CL) to implement in order to protect the public health and the environment. CLs consist in a combination of technical characteristics of the facility, safety equipment, laboratory practices and operational procedures [15]. Generally, facilities, including laboratories, animal housing areas and production facilities are classified into four CL ascending from CL-1 to CL-4 [15,19], in which CL-4 implements the most stringent containment measures in order to contain extremely virulent and transmissible micro-organisms during their manipulation. The risk assessment is specific for an activity, therefore additional containment measures can be required to the standard measures defined by a given containment level.

When the GMO vaccine candidate (also named recombinant vaccine candidate) has achieved pre-clinical studies without any obvious adverse effect and with proven protective efficacy in different animal models, it enters the clinical stage of development in humans. Clinical studies cover four stages over several years, from initial phase I clinical trials in humans assessing the safety in healthy humans to phase III trials assessing efficacy against the disease under natural disease conditions and finally post-marketing surveillance to detect adverse effects as well as to assess long term efficacy (phase IV). Clinical development is built on rigorous ethical principles of informed consent from volunteers or patients, with an emphasis on vaccine safety as well as efficacy. Clinical trials using recombinant vaccines, as all human clinical trials performed in the EU, fall under the scope of Directive 2001/20/EC establishing provisions regarding the conduct of clinical trials on human subjects involving medicinal products (Table 1) [18].

In the framework of the European biosafety directives, at each clinical stage, Directive 2001/18/EC on deliberate release of GMOs must be considered in case the vaccine candidate enters into contact with the environment and the general population. Dissemination of a recombinant vaccine into the environment is not an adverse event *per se* but an ERA must be performed with the aim of evaluating (i) the potential of the GMO to cause adverse effects on persons (other than treated subjects), animals, plants and other micro-organisms exposed to it and (ii) the probability that these adverse effects will occur. The ERA requires an assessment of the genetic stability of the recombinant vaccine and the possible interaction of the GMO with other organisms [20]. It has to take into account the intrinsic characteristics of the strain used, the characteristics of the transgene(s), the biodistribution and level of dissemination of the GMO, the possibility of recombination and the risk classification. Indeed, a critical step in the ERA is evaluating pathways of exposure through which the recombinant vaccine may interact with humans (other than vaccinee) or the environment. It excludes pure medical aspects on the efficacy and safety of the vaccine for the treated subject, even though data on safety are very useful to assess the potential pathogenicity of the vaccine candidate and may inform the ERA. It is worth mentioning that the exposure of the general population and the environment will usually be lower than the exposure of the vaccinee [21].

## 3. Update of TB Vaccine Candidates in Clinical Trials

Research and development of new and effective vaccines has increased in the last 20 years after recognition of the limited effectiveness of BCG as a global vaccine approach and the increasing incidence of MDR-TB impacting upon chemotherapy-based TB control programs. The ideal TB vaccine would be a pre-exposure vaccine that is able to elicit sterilizing immunity preferably at the time of initial infection. Nevertheless, given the complex interactions between *Mtb* and its host and the poor understanding of the correlates of protection, current vaccine research rather aims at developing a vaccination protocol impacting on the transmission of the infection by inducing levels of immunity that are able to prevent the development of active TB, to be administered before *Mtb* exposure or to latently infected individuals.

TB vaccines under development fall under two main categories. The first category is the one of vaccines that could replace BCG. Indeed, as BCG is protective against childhood forms of TB, a new vaccination protocol should involve a vaccine that is able to prevent these forms of the disease and that could be administered to newborns as it is currently done with BCG. The second category is the one of sub-unit vaccines to be administered as booster vaccines later in life to individuals that have been vaccinated with BCG during infancy and that should prevent the development of adult pulmonary TB. These booster sub-unit vaccines are also tested in individuals latently infected with *Mtb*. The rationale behind all the booster vaccines is the notion that BCG induced immunity wanes with time, although this notion is not accepted by everyone in the scientific community [22,23]. It was already demonstrated that homologous boosting with BCG is not protective [24], while pre-clinical data indicate that using sub-unit vaccines consisting of protective mycobacterial antigens can improve protection [25].

Several TB vaccine candidates are currently at different stages of clinical trial development (phase I to phase III). Designed as BCG replacements are two GMOs: a genetically modified BCG strain called VPM1002 and a genetically modified *Mtb* strain called MTBVAC. Designed as booster vaccines are the viral-vectored sub-unit vaccines MVA-85A, MVA-85A-IMX313, AdHu5Ag85A, AERAS-402 and ChAdOx1 85A based on Modified Vaccinia virus Ankara (MVA) or on modified adenoviruses (Ad). Different recombinant proteins (genetically engineered fusions of mycobacterial antigens) are also currently in clinical development. These proteins are formulated in adjuvants such as the M72, fusion of Rv0125 and Rv1196 formulated in the liposome-based AS01 adjuvant system; Hybrid1 (a fusion of Ag85B and ESAT-6), Hybrid56 (a fusion of Ag85B, ESAT-6 and Rv2660) or Hybrid4 (a fusion of Ag85B and TB10.4) formulated in the adjuvant IC31 and finally the ID93 fusion of four *Mtb* antigens linked in tandem (namely Rv3619, Rv1813, Rv3620 and Rv2608) formulated in GLA-SE (Glucopyranosyl lipid adjuvant-stable emulsion), a synthetic adjuvant based on a TLR4 agonist [26,27,28,29,30].

In this review, we will focus on the genetically modified mycobacterial strains (MTBVAC and VPM1002) and the viral-vectored sub-unit vaccines that fall under the category of GMOs (Table 2). Issues related to the other sub-unit vaccines in clinical development, which involve the use of GMOs for their development and production, will not be discussed here.

**Table 2:** GMO-based vaccine candidates against TB in clinical trials.

Vaccine	Backbone	Genetic modification	Current clinical phase of development	Ref.
GMO-based vaccine candidates designed to replace BCG				
VPM1002	Recombinant BCG	*ΔureC::hly* Hm(R) deleted in *ureC* expressing listeriolysin (*hly*) from *Listeria monocytogenes*	Phase II	[31,32]
MTBVAC	Recombinant *Mtb*	deleted in *phoP* and *fadD26* without antibiotic-resistance markers	Phase I	[33]
Viral vectored sub-unit vaccines designed as booster vaccines				
MVA-85A (also called AERAS-485)	Recombinant Vaccinia Ankara vector	Expressing *Ag85A* (*Rv3804c*)	Phase IIb	[34,35,36,37,38,39,40,41,42,43,44,45,46,47]
MVA-85A-IMX313	Recombinant Vaccinia Ankara vector	Expressing a fusion of *Ag85A* (*Rv3804c*) and IMX313	Phase I	[48]
AERAS-402 (also called Crucell Ad35)	Recombinant replication deficient Adenovirus serotype 35 (Ad35)	Expressing *Ag85A* (Rv3804c); *Ag85B* (Rv1886c) and *TB10*.*4* (Rv0288) as a fusion protein	Phase II	[49,50]
		Deleted in E1		
AdAg85A	Recombinant replication deficient Adenovirus serotype 5 (Ad5)	Expressing *Ag85A* (Rv3804c)	Phase I	[51,52]
		Deleted in E1 and E3		
ChAdOx1 85A	Recombinant replication deficient simian Adenovirus	Expressing *Ag85A* (Rv3804c)	Phase I	[53]
		Deleted in E1 and E3		

## 4. Biosafety Considerations of Clinical Studies with GMO-Based Vaccines

Human use of a GMO as vaccine requires rigorous tests to control their attenuation and the absence of reversion to virulence, their persistence and biodistribution in the vaccinated subject, their ability to survive outside the host in the environment and their genetic stability [54,55]. Issues raised by use of antibiotic resistance markers need to be addressed and evidence for lack of shedding of live micro-organisms by vaccinated persons should also be assessed. Concerning recombinant live mycobacteria, the possibility of exchange of genetic material with naturally occurring environmental mycobacteria needs to be studied and requires an assessment of the consequences of such genetic exchange.

### 4.1. BCG Replacement with Genetically Modified Mycobacteria

One of the basic strategies to develop this first category of TB vaccines is to improve BCG by genetically modifying it. The resulting genetically modified (GM) BCG strain will express or over-express *Mtb* protective antigens or will be able to better induce protective immune responses as compared to the original BCG vaccine strain. The second strategy followed to replace classic BCG vaccination is rational attenuation of *Mtb* in order to develop a live attenuated TB vaccine that resembles more the pathogenic mycobacteria in terms of antigenic repertoire and in terms of the induced immune responses.

#### 4.1.1. Genetically Modified BCG in Current Live Vaccine Candidates: VPM1002

BCG was developed by attenuation of virulent *Mycobacterium bovis* through 230 passages in glycerin-bile-potato medium over the course of 13 years [56]. The original BCG vaccine was distributed to laboratories that have continued passaging the strain and consequently new sub-strains of BCG arose with somewhat different genetic background. The various sub-strains are divided into two broad groups known as early and late strains [57]. They show genetic variations but no substantial differences were observed between them in efficacy and protection against *Mtb* in guinea pigs [54,58]. All BCG vaccine strains have lost RD1 (Region of Difference 1) encoding the machinery required to synthesize and export the major T-cell antigen/virulence factor ESAT-6/CFP-10. Deletion of RD1 is essential for attenuation of virulent *M*. *bovis*.

Taking advantage of the information accumulated from BCG studies over the years, the vaccine strain BCG is being widely used as a backbone to design improved recombinant BCG derivatives as vaccine against TB. Two strategies have been adopted to improve vaccine efficacy over parental BCG: (i) by designing means to induce increased antigen-specific responses by favouring antigen escape from the phagosomal compartment and increase cross-presentation of mycobacterial antigen over those achieved by parental BCG or (ii) by constructing BCG strains that are able to over express protective antigens.

Three GMO vaccine candidates based on BCG entered in clinical trials: VPM1002, AERAS-422 and rBCG30 [31,59,60]. VPM1002 is the only one that successfully continues in phase II clinical trial, the two others have been stopped, in the case of AERAS-422 for possible safety reasons (some trial participants developed shingles). Other promising candidates using BCG as a backbone are still in pre-clinical stage [61,62].

The vaccine candidate VPM1002 is a GM BCG Danish subtype Prague strain expressing the listeriolysin protein (LLO) and carrying a urease gene deletion [31]. VPM1002 has been tested in two phase I trials, one in Germany and one in South Africa, and a phase II trial to evaluate its safety and immunogenicity in comparison with BCG in newborn infants was recently completed in South Africa (NCT01479972). The rationale behind the development of this recombinant BCG strain was to overcome the poor ability of BCG to induce CD8^+^ T cell responses by integrating into its genome the gene *hly* of *Listeria monocytogenes*. LLO, the protein encoded by *hly*, is responsible for the formation of pores in the phagosome after endocytic uptake of the bacterium, which might enable VPM1002 to access the cytosol and result in MHC class I presentation of BCG derived peptides.

A phase I clinical trial with VPM1002 vaccine candidate has been authorized and achieved on healthy volunteers in Germany. A thorough risk assessment of the trial has been performed as described in European Directives on the contained use and the deliberate release of GMO. A summary of this analysis is now publicly available in an SNIF (summary notification information format) [63].

##### 4.1.1.1. Characteristics of the Parental BCG

According to international classification lists, BCG may belong to RG 2 (in UK and USA) or RG 1 (in Switzerland and Germany) [64,65,66,67]. BCG is considered as a safe vaccine and has been administered to hundreds of millions of neonates in the world without serious side effects. However, on rare occasions BCG may cause abscesses at the site of inoculation and localized lesions such as osteitis. The most serious complication of BCG is disseminated disease that results from impaired immunity [68]. BCG administration is thus contraindicated in HIV infected infants as it may pose a considerable risk of distant or disseminated BCG disease [69,70].

In a laboratory or clinical setting, workers manipulating BCG may be exposed to this agent through contact of mucous membranes or injured skin to aerosols or droplets containing the organism but also by an accidental inoculation or ingestion of material containing BCG [67]. Mycobacteria show generally high stability and capacity to persist outside a host into the environment as well as a high resistance to dehydration [71]. However, these micro-organisms present slow replicative rates and therefore a poor propagative capacity. A CL-2 is generally required for manipulations of BCG in a laboratory and workers should wear as a minimal personal protective equipment: a lab coat, gloves and safety glasses [67,72]. In addition, all aerosol-generating activities must be conducted in a class II Biosafety Cabinet (BSC).

The genetic background used in VPM1002 is the BCG Danish, subtype Prague. In this sub-strain, BCG has lost the genetic segments RD1 and RD2 carrying genes implicated in virulence [73,74].

##### 4.1.1.2. The Transgene and the Genetic Modification

In VPM1002, BCG expresses a recombinant fusion protein composed of the leader sequence of the mycobacterial antigen 85B in front of LLO, a secreted thiol-activated cholesterol-binding hemolysin showing hemolytic activity around pH 5.5. In order to guarantee the optimal pH for listeriolysin activity the gene *ureC* encoding the mycobacterial urease C sub-unit was disrupted by insertion of the Ag85B-LLO expression cassette [31]. The *hly* expressing cassette is placed under the regulation of the mycobacterial promoter sequence from the heat shock protein 65 (hsp65).

LLO is a toxin considered as a virulence factor of the bacterium *L*. *monocytogenes*, the causative agent of listeriosis. As already mentioned, the mechanism by which VPM1002 might improve immunogenic protection against TB compared to parental BCG is that perforation of the membrane surrounding the phagosome by LLO facilitates translocation and subsequent MHC I loading of mycobacterial antigens [75]. In addition, studies in mouse and human macrophages reported that phagosomal membrane perforation might activate cell apoptosis following leakage of phagosomal enzymes in the cytosol which in turn results in cross-priming of the mycobacterial antigens [76]. Indeed, *Hly* from *L*. *monocytogenes* has been shown to induce apoptotic cell death in different cell types [77,78].

LLO construction in VPM1002 provides two safety features in order to limit perforation to phagosome membrane of the infected cell: the pore-forming function of the protein is restricted to acidic pH and LLO carries the PEST amino acid sequence (containing proline, glutamic acid, serine and threonine) that directs the protein to phagosomal degradation immediately upon appearance in the host cell cytosol [79,80]. In pre-clinical studies on Rhesus monkeys, no indications of lysteriolysin induced hemolysis were reported after an intradermal injection with the research precursor of VPM1002 (rBCG∆ureC::Hly^+^::Hyg^+^) [81].

pVEP2003, the plasmid used to generate the VPM1002 is an *E*. *coli* shuttle vector, based on the pJSC284 plasmid. The plasmid carries an *E*. *coli* origin of replication but no mycobacterial origin of replication, which makes this plasmid a suicide plasmid which cannot replicate in mycobacteria [63]. The plasmid carries no transposable elements. Beside the *hly* expressing cassette, the vector contains a hygromycin resistance cassette for selection reasons [32]. It is flanked by 2 γδ resolvase recognition sites used to excise the hygromycin out of the VPM1002 vaccine candidate upon extrachromosomal expression of the resolvase. However, it is not clear from available data if this marker is removed from VPM1002. It is worth mentioning that generally, the use of antibiotic resistance genes as markers should be limited as much as possible in order to prevent potential risks associated with exposure of humans and the environment to these markers.

##### 4.1.1.3. Genetic Stability of VPM1002

Hundreds passages of *M*. *bovis* have resulted in different BCG strains and sub-strains demonstrating genetic variability of the micro-organism. However, no other factors of genetic modification have been reported since the initial use of BCG except the general mutational factors (drugs or radiation). History of parental BCG practical use as vaccine during years shows no evidence of gene reversion to virulence or gene complementation. It does not reveal any evidence of horizontal gene transfer capacity of BCG [20]. During construction of VPM1002, the insert is integrated in the bacterial chromosome, making horizontal gene transfer highly improbable. Research on VPM1002 reports that the vaccine shows genetic stability after several laboratory passages [63]. Reproducible conditions of culture and manufacturing of the vaccine would indeed maintain stability of the VPM1002 genome. For the use in vaccination studies on humans, genetic stability of the recombinant BCG material need to be confirmed which means that VPM1002 material has to be well-characterized and the genetic modification has to be confirmed by molecular techniques such as PCR, Southern blotting and sequencing [55].

##### 4.1.1.4. Safety of VPM1002

Pre-clinical and clinical studies bring valuable information concerning safety of VPM1002 vaccination compared to BCG vaccination which can be used to assess the potential pathogenicity of the GM vaccine [31,32,55]. VPM1002 was shown to cause no serious adverse effects in different animals used as models for different immunological status (IFNγ knock-out mice and SCID mice) and age (newborn rabbits) [55]. In rhesus monkeys vaccinated with the precursor of VPM1002, no other adverse effect than a mild local reaction such as that observed in BCG vaccinated animals was reported [81]. The report on the first results of phase I clinical studies in human confirms safety of VPM1002 on healthy volunteers from Germany and South Africa [32].

##### 4.1.1.5. Transmission of VPM1002

No studies seem to have reported specific information on VPM1002 transmission modes. During manipulations in a laboratory or a clinical setting, VPM1002, as a mycobacterium, may transmit to workers by inhalation of infected droplet nuclei, by contact with non-intact skin and mucous membranes and by accidental inoculation or ingestion of infected material. Nevertheless, as the precursor of VPM1002 has showed lower persistence in infected mice macrophages than the parental BCG, the probability of transmission to other organisms is considered lower as well [75,82]. In the framework of surveillance of unintended transmission of the vaccine during the phase I clinical studies, no suspected transmission of the vaccine to other persons has been reported [32].

##### 4.1.1.6. VPM1002 Risk Classification

The risk classification of VPM1002 depends on the nature of the parental organism (BCG), the transgene and the consequences of the insertion on the parent characteristics. BCG has been classified in RG 2 or RG 1 depending on the international classification list considered. Effect of LLO, the product of *hly* expression in VPM1002, combined to the urease gene deletion, appears directed specifically to the phagosome membrane and suggests that the transgene does not potentiate risks linked to the use of VPM1002. Indeed, no deleterious effects have been observed after administration of this vaccine candidate to animals and humans in preclinical and clinical studies, suggesting that VPM1002 is not pathogenic. Inventors even underline that the rate of adverse events of VPM1002 vaccination was lower than the parental BCG vaccination [63]. Also, compared to the parental BCG, VPM1002 has been shown to be more rapidly degraded in the organism. Finally, it has to be mentioned that VPM1002 is sensitive to antibiotics commonly used in the treatment of mycobacterial infection (e.g., isoniazid, rifampicin and ethambutol) [55,63].

Taking into account these characteristics of VPM1002 and the first results of clinical studies, this vaccine candidate may be classified in RG 1, namely, a biological agent with no or a negligible risk for human health and the environment and for which a CL-1 is required.

##### 4.1.1.7. Biodistribution of VPM1002 and Environmental Risk Assessment

VPM1002 has been shown to have a low multiplication rate and a weak persistence in infected cells [75,82]. No viable bacilli in slices of lungs, livers and lymph nodes of guinea pigs were detected by culture after vaccination with VPM1002, though PCR data revealed that a systemic spread of VPM1002 had occurred in these rodents [55]. This observation suggests that excreted biological fluids of a vaccinated subject may contain DNA of the recombinant BCG which may by this way be released into the environment. In the framework of clinical trials, putative routes of transmission of the vaccine were explored through the analysis of blood, saliva, urine and stool from vaccinated volunteers for traces of vaccine (PCR analysis of unique genomic DNA regions of VPM1002). All results were reported negative and no suspected transmission of the vaccine to other persons were observed during the first clinical phases [32,63].

Shedding of VPM1002 was not detected as reported by analysis of body fluids from vaccinated subjects. The probability of VPM1002 dissemination into the environment from the vaccinated person is therefore considered very low. However, if the vaccine candidate spreads into the environment, the supposed genetic stability of VPM1002 is foreseen to limit its interaction with other mycobacteria. The possibility of exchange of genetic material with naturally occurring environmental mycobacteria needs to be studied and requires an assessment of the consequences of such genetic exchange.

If an external, non-vaccinated person such as a clinical worker or a relative is contaminated by VPM1002 no toxic effects are foreseen from the newly introduced gene. The first clinical trial with VPM1002 in humans compared the number and intensity of adverse effects (AE) in BCG-naïve volunteers and in BCG-vaccinated persons. Although the number of AE was higher in the BCG-vaccinated group, their intensity was similar to those in the group of BCG-naïve subjects (mostly mild AE), suggesting that VPM1002 may not induce any exaggerate immune response (Koch phenomenon) in a person already vaccinated with BCG. Similarly, it might be assumed that the same situation will occur in a person (other than the vaccinee) who is latently infected with *Mtb* and who comes inadvertently into contact with the vaccine candidate. However, this assumption needs to be confirmed. At the opposite, an unintentional exposure to VPM1002 of a human who is immunosuppressed or HIV infected may probably increase the risk for this person to develop BCG complications. Indeed, BCG vaccination is not recommended in HIV-infected infants and in adults.

##### 4.1.1.8. Risk management Measures (Containment, Worker Protection Measures, Waste)

The high attenuation profile of VPM1002 allows handling of this GMO under a CL-1 (Directive 2009/41/EC). However, this level is appropriate to manipulate VPM1002 at the condition that the vaccine candidate has been well-characterize before use in humans. Additional measures should be taken to prevent or manage risks associated with manipulations by the clinical personnel and with dissemination into the environment of VPM1002 (Table 3). Transmission to workers may occur by inhalation of the airborne VPM1002, by an inadvertent exposure of mucous membranes or damaged skin to contaminated aerosols, droplets or materials or by an accidental parenteral inoculation with a needle or a sharp instrument carrying VPM1002. These potential incidents involving the clinical personnel may be at the origin of an unintentional release of the GMO vaccine candidate into the environment. The prevention of VPM1002 exposure of personnel during manipulations of VPM1002 material will consist for workers, in the application of specific work practices and in wearing personal protective equipment. In particular, all open phase operations with VPM1002 material should be carried out in a class II BSC and workers should limit as much as possible the use of sharp objects. Adapted procedures should be applied for material and surface decontamination taking into account the properties of mycobacteria (high survival capacity outside the host and resistance to some standard disinfectants). Potentially contaminated waste and personal protective equipment should be inactivated using an appropriate method before disposal.

**Table 3:** Work practices for personnel manipulating genetically modified TB vaccines to prevent or manage risks for public health and/or the environment.

Aerosol producing operations should be reduced during preparation and administration of the GM vaccines and personnel manipulating the GM vaccines should wear adequate protective clothing such a lab coats, goggles. Some manipulations should preferably be carried out in a class II biosafety cabinet. Work with needles and other sharp objects should be strictly limited and workers should never recap nor remove needles from syringes. Removal of the needle from the syringe should occur by means of hand free operations (*i.e*., hands do not touch the needle) into a closed container. The use of gloves is required to avoid skin contamination. Appropriate procedures for material and surfaces decontamination should be applied. Spills should be inactivated by an appropriate disinfectant, allowing sufficient contact time before disposal. Contaminated waste and personal protective equipment should be inactivated using an appropriate method before disposal. Potentially contaminated non-disposable materials need to be properly decontaminated. If an incident occurs that could lead to infection (e.g., breakage of a vial containing the GM vaccine, or needle stick), applicable first aid should be performed (*i.e*., flushing eye for ocular exposure, placing an absorbent tissue on the affected area in order to absorb all viral particles and apply disinfectant directly to the tissue for percutaneous exposure), followed by reporting to the supervisor. The injection site should be protected and covered. Appropriate waste management measures and disposal should be taken by the patient.

Finally, the volunteer who has been vaccinated with VPM1002 and goes back home should take measures to protect the injection site to avoid direct contact of the vaccine with any other person and to limit dissemination of the GMO into the environment. In the case of VPM1002, as for BCG, the vaccine is administered intradermally to the subject. This route of administration is recommended for several reasons: (i) a more consistent dose is delivered to the patient than for other routes of administration; (ii) the skin presents a dense network of immune-stimulatory antigen-presenting cells increasing the immunogenicity of the vaccine and (iii) the intradermal route reduces the risk of needle-stick injuries for caretakers and the risk of blood or nerve injuries for patients. However, compared to intramuscular injection, intradermal administration of a vaccine results also in a potential increase of local adverse events and in a superficial lesion that may last three months after vaccination in the case of BCG. Lesions at the injection site become a way of vaccine leakage into the environment. Thus the VPM1002 injection site should be covered at least until complete healing of the lesion. In addition, it is important to consider adequate management and disposal of the waste generated by the vaccinee during this period.

#### 4.1.2. *Mtb* Genetic Background of Current Live Vaccine Candidates: MTBVAC

The second strategy that is followed in order to develop a BCG replacement is the rational attenuation of *Mtb* by genetically modifying it. As cured TB does not prevent reinfection, the success of this approach is unpredictable. On the other hand, PPD (Purified Protein Derivative) positive subjects who were exposed to *Mtb* and developed a latent TB infection can control the infection in 90%–95% of the cases. The resulting attenuated strain will resemble more the pathogenic mycobacteria in terms of antigenic repertoire and in terms of induced immune responses than the *M*. *bovis* derived BCG vaccine. The biosafety issues need to be carefully addressed as these attenuated strains are derived from a pathogen belonging to the RG 3 micro-organisms, namely a biological agent that can cause a severe disease and that presents an elevated risk of spreading to the community. Thus, as a minimal requirement for approval for human use, an *Mtb*-based vaccine candidate needs to comply with the Geneva Consensus calling for at least 2 unlinked non-reverting mutations in *Mtb* vaccine strains and the absence of an antibiotic resistance gene [20]. So far only the MTBVAC candidate is being tested in a phase I clinical trial after a long procedure to obtain regulatory approval for clinical safety testing. The aim of this trial is to assess the safety and immunogenicity of MTBVAC in a dose escalation study. According to MTBVAC investigators, the preliminary safety results are already promising [83]. Immunogenicity results are expected for 2014.

##### 4.1.2.1. Characteristics of the Parental *Mtb*

*Mtb* is a highly communicable human pathogen. It has a very low infectious dose and transmission may occur through contaminated objects or surfaces or by inhalation of droplet nuclei carrying *Mtb*. Other modes of transmission, albeit less frequent, include percutaneous transmission such as direct injury to the skin and mucous membranes through breaks in skin [3]. Infected animals (but not mice) can spread the infection to laboratory workers through aerosols, fomites or animal bites. Risk of transmission is increased by the capacity of *Mtb* to persist in the environment for long periods. In R&D facilities, a CL-3 facility is required for work involving infectious or potentially infectious materials, animals or cultures.

##### 4.1.2.2. Genetic Modifications in MTBVAC

MTBVAC is an *Mtb* strain deleted in the transcriptional regulator *phoP* and in *fadD26* [33]. These two steps mutations were engineered on a human clinical isolate of *Mtb*, namely MT103. *PhoP* is a transcriptional regulator part of a 2-component system involved in sensing and adaptation of the pathogen to an intracellular environment. It has been proposed to constitute a key regulator in the control of *Mtb* growth-associated virulence [84,85]. Its expression was shown to be strongly upregulated in a clinical isolate demonstrated to be a cause of MDR-TB [86], indicating that PhoP is a transcription factor contributing to virulence. Indeed, *Mtb∆phoP* cannot express numerous genes including important virulence factors [87].

The second deleted gene in MTBVAC is *fadD26* whose gene product is required to synthesize phtiocerol dimycocerosates (PDIMs). PDIMs are unique components of the mycobacterial cell wall playing an important role as a permeability barrier and in pathogenicity of mycobacteria from the *Mtb* complex [88,89]. PDIMs have been shown to protect the intracellular pathogen from host defense and to be required for multiplication in mouse lungs [90].

Plasmids used to genetically delete *phoP* and *fadD26* in MT103 *Mtb* strain consist in two suicide vectors carrying *phoP* or *fadD2*6 genes disrupted by insertion of an antibiotic resistance marker cassette. Each insert (*fadD26::Ώhyg* and *phoP::Ώkm* ) was incorporated into a pJQ200X vector used to achieve their insertion in *Mtb* genome by homologous recombination. This clonal vector is a suicide vector that cannot replicate in mycobacteria. The antibiotic resistance markers are flanked by 2 γδ resolvase recognition sites that are used to excise the antibiotic genes out of the vaccine candidate in presence of resolvase, leaving only res sites in the final MTBVAC [91,92].

##### 4.1.2.3. Genetic Stability of MTBVAC

At the second Geneva meeting on recommendations to follow for novel live TB vaccines clinical studies, it was globally accepted that mycobacteria and their GM derivatives are genetically highly stable with a lack of evidence of gene reversion complementation or horizontal gene transfer [20]. Large clinical and practical experience with BCG vaccine supports this opinion. In the case of MTBVAC, inserts are integrated in the bacterial chromosome, decreasing horizontal gene transfer probability. However, the possibility of exchange of genetic material of recombinant *Mtb* with naturally occurring environmental mycobacteria and the consequences of such genetic exchange should be explored, particularly to assess the probability of attenuation complementation.

As mentioned earlier for VPM1002, reproducible conditions of culture and manufacturing of the vaccine would maintain stability of the MTBVAC genome. Before its use in vaccination studies on humans, genetic stability of the recombinant *Mtb* material has to be checked and the vaccine candidate has to be well characterized.

##### 4.1.2.4. Safety of MTBVAC

Both mutations in MTBVAC affecting key virulence regulators of *Mtb* have resulted in *Mtb* attenuation. Safety studies have been performed earlier for each individual genetic modification that result in MTBVAC vaccine in animal models. Investigators have shown that an *Mtb* mutant strain that has the *phoP* gene inactivated (SO2 strain) is more attenuated than the BCG strain [87]. The same observations are reported for the recombinant *Mtb* that has the *fadD26* gene inactivated [93]. Both individual *Mtb* mutants are also reported to provide protection levels higher or comparable to those conferred by BCG in mice. The mutant *Mtb* strain MTBVAC with the combination of the 2 independent mutations, one in synthesis of the PhoP protein and one in PDIM synthesis, has been shown to be more attenuated than BCG in SCID mice after intravenous inoculation of the vaccine candidate [92]. Toxicity studies with guinea pigs inoculated with a 50 times the BCG vaccine human dose, showed that these animals gained weight over a period of six months and no visible TB lesions were observed on these animals at autopsy [33,92]. It is also reported to provide a greater degree of protection than BCG in the guinea pig model [33]. So far, no data are already available on the safety of MTBVAC vaccination in human volunteers.

##### 4.1.2.5. Transmission of MTBVAC

No specific data on transmission modes of this vaccine candidate can be found in the literature. The genetic modifications of *Mtb* in MTBVAC may not modify the transmission characteristics of the parental *Mtb* strain. The recombinant *Mtb* may then transmit by inhalation of infected droplet nuclei. Indeed, SCID mice have been inoculated by aerosol with MTBVAC in studies of vaccine attenuation [92]. Other ways of transmission include contact of MTBVAC aerosols with damaged skin and mucous membranes and an accidental parenteral inoculation of MTBVAC material. It is worth mentioning that the recombinant *Mtb* presence has been shown to decline after vaccination in mice decreasing as such the probability of transmission from the vaccinee. The capacity of MTBVAC to persist outside a host may be high and similar to that generally shown by mycobacteria, or may be altered as a consequence of deletion of *phoP* or *fadD26* genes. This still needs to be determined. Similarly it is not well established if genetic modifications in MTBVAC may alter the growth rate of the *Mtb* double mutant.

##### 4.1.2.6. MTBVAC Risk Classification

The parental *Mtb* strain of MTBVAC is classified in RG 3. In MTBVAC, two independent deletions in *fadD26* and *phoP* genes affecting key virulence factors have attenuated the strain as demonstrated in pre-clinical studies on MTBVAC. The safety profile of MTBVAC has been reported to be better than the BCG profile in immune-compromised SCID mice [33]. In addition, the *Mtb* double mutant remains sensitive to antituberculosis drugs, which would allow a conventional treatment. As already mentioned, at the Geneva Consensus meeting on the safety requirements for novel TB vaccine candidates, participants claimed that mycobacteria and their GM derivatives are genetically stable with any evidences of gene reversion complementation or horizontal gene transfer. Thus, nowadays and taking into account results reported by pre-clinical studies, MTBVAC would be classified in RG 1 for animals. Data are missing to determine a final risk group of MTBVAC in humans. Results from the phase I clinical trial in humans are expected to provide valuable information to be used in a thorough risk assessment of MTBVAC.

##### 4.1.2.7. Biodistribution of MTBVAC and Environmental Risk Assessment

MTBVAC biodistribution in mice was reported to be localized mainly in lymph nodes where a progressive clearance of the colonies was observed after four weeks of intradermal vaccination [33]. MTBVAC could not be detected in urine and stool of mice. No information has been found on its presence in saliva or blood. In guinea pigs, viable MTBVAC was exclusively found in the site of vaccination only immediately after intradermal administration.

Vaccine shedding was not detected in urine and stool of MTBVAC inoculated mice thereby indicating that the probability of MTBVAC dissemination into the environment from a vaccinated animal may be low. This should be assessed in humans during the first phase clinical study using MTBVAC. In case of vaccine candidate release into the environment, only a limited environmental impact is expected to occur if MTBVAC has kept the poor replicative characteristics and the high genetic stability of mycobacteria. However, the possibility of genetic exchange occurrence with environmental mycobacteria may exist and should be explored mainly to discard any possible reversion of MTBVAC to virulence. Similarly, no adverse effects of the attenuated MTBVAC are anticipated in a person (other than the vaccinee) coming into contact with this vaccine candidate. Nevertheless and as discussed for VPM1002 vaccine candidate, an exposure to MTBVAC of an external person latently infected with *Mtb* or previously vaccinated with BCG may induce a severe immune reaction. This should be explored in current clinical study with MTBVAC.

##### 4.1.2.8. Risk Management Measures (Containment, Workers Protection Measures, Waste)

MTBVAC may be handled in a CL-1 facility with some additional measures (Directive 2009/41/EC; Table 3) taking into account the risk assessment of MTBVAC in animals and considering that the genetic characteristics and stability of MTBVAC have been confirmed before use in the clinical setting. Transmission to personnel manipulating the vaccine could occur by inhalation of the airborne MTBVAC, by exposure of mucous membranes or damaged skin to droplets, aerosols containing the recombinant *Mtb* and by an accidental parenteral inoculation when handling a needle or any sharp object carrying MTBVAC. Prevention of worker exposure and release of MTBVAC into the environment should be managed by use of adapted equipment such as a BSC for open phase manipulations of the MTBVAC material and by bearing adequate personal protective equipment. Workers should limit as far as possible the use of sharp objects. Surface decontamination, waste inactivation and disposal methods should be adapted to mycobacteria characteristics. At home, appropriate measures should be taken by the vaccinee to cover and protect the site of injection. This measure aims to limit vaccine dissemination from the site of administration and vaccine contact with external persons particularly if the recombinant vaccine is injected intradermally. As already mentioned, this route of administration may result in a local superficial lesion that may be the origin of an unintended release of the recombinant *Mtb* into the environment. Likewise, potentially contaminated waste generated by this procedure should be collected and inactivated before adequate disposal.

In the framework of MTBVAC phase I clinical trial on humans, investigators also recommended to volunteers to avoid as far as possible closed contacts with old or young people or persons who are immune-compromised or HIV infected during the study period (7 months). Indeed, an exposure to MTBVAC may increase the risk for these persons to develop a TB related disease.

These measures should be evaluated and adapted if necessary in function of the results and information obtained from the ongoing or future studies of the GM *Mtb* vaccine in humans.

### 4.2. TB Vaccine Candidates Based on Recombinant Viral Vectors as “Booster” Sub-Unit Vaccines

Another category of TB vaccines under development is the one of preventive TB “booster” sub-unit vaccines, which have been conceived to be administered in a vaccine regimen involving BCG vaccination at birth followed by a boost vaccination. These booster sub-unit vaccines are also tested in individuals latently infected with *Mtb*. The rationale behind all the booster vaccines is the notion that BCG induced immunity wanes with time. BCG-induced protective efficacy is thought to last 10–15 years but at least one study has reported on a much longer persistence of protection [22,94]. The following viral-vectored candidates are currently tested in clinical trials: AERAS-402, AdHu5Ag85A, ChAdOx1 85A, MVA-85A and MVA-85A-IMX313 (Table 2).

AERAS-402 is a recombinant replication deficient Adenovirus serotype 35 (Ad35) vector expressing the mycobacterial antigens Ag85A, Ag85B and TB10.4 as a fusion protein. AERAS-402 has been tested in early-stage clinical trials in healthy adults and in adults with recently treated pulmonary tuberculosis [49,50]. It has also been evaluated in a phase IIb in infants vaccinated with BCG as well as in HIV infected adults, with latent TB and prior to BCG vaccination. Two trials are ongoing, one on infants and the other on adults in combination with the MVA recombinant vector MVA-85A described hereafter.

AdHu5Ag85A (AdHu5 for human Ad5) is a recombinant replication deficient Ad5 vector expressing the mycobacterial antigen Ag85A [52]. The safety and the immunogenicity of AdHu5Ag85A vaccine has been demonstrated in healthy humans in a phase I clinical study [51]. Pre-existing anti-AdHu5 antibodies have a minimal negative effect on Ag85A-specific T cell reactivity in healthy adult humans and interestingly did not affect the safety and efficacy of the vaccine [95].

Replication defective Ad have been employed extensively as vaccine because they induce a strong humoral and especially a strong T cell response, skewed towards T helper type 1 immunity specific for the antigen expressed by the vector [95].

Most Ads naturally infect the respiratory tract and can be transmitted by direct contact, faecal-oral route or respiratory droplets [96]. They are usually acquired in early childhood and cause infections of the upper respiratory tract and to a lesser extent the gastrointestinal and urinary tracts. Human Ads are divided into 7 species and 55 serotypes [95]. Wild-type Ad35 belongs to species B and has a tropism for the urinary tract. Wild-type Ad5 belongs to species C and has a tropism for the respiratory tract. Human Ads are ubiquitous, and most people have been infected with one or more serotypes, leading to lifelong immunity. Most adults have been exposed to Ad5. At the contrary, Ad35 is rare in the human population and seroprevalence and levels of neutralizing antibody titer to Ad35 in adults are much lower than those of Ad5 (seroprevalence: 20% *vs*. 90%, respectively) [49].

Adenovirus infection can be asymptomatic or can lead to disease which is usually mild in immunocompetent individuals. Whereas most Ad infections are mild, Ads can be dangerous in immunosuppressed individuals, especially transplant patients in which Ads probably reactivate from latent or low grade persistent infections [97]. Wild-type human Ads belong to RG 2. Ad are relatively stable, resistant to dehydration, able to persist in aerosols and water.

MVA-85A (also called AERAS-485) is a recombinant of the Modified Vaccinia Ankara (MVA) strain expressing Ag85A. Several phase I and II clinical trials have been conducted and are still ongoing with the MVA-85A. The trials performed so far mostly concern humans (infants, children and adults), but MVA-85A has also been tested for vaccination of calves against bovine TB. Noteworthy, MVA-85A is also tested in individuals that are latently infected with *Mtb* as well as in HIV infected patients.

Recently, a phase I clinical trial with MVA-85A-IMX313 has started (NCT01879163). MVA-85A-IMX313 expresses the fusion of Ag85A with IMX313, the oligomerization domain encoded by the last exon of the complement 4 binding protein (C4bp) alpha-chain derived from chicken [48]. The aim of this phase I trial currently recruiting participants is to evaluate the safety and immunogenicity of MVA-85A-IMX313 vaccination compared to MVA-85A vaccination in BCG vaccinated adults. Finally, it is worth mentioning that there are also plans to administer subsequently at a 3-month interval the 2 booster vaccines AERAS-402 and MVA-85A (NCT01683773).

The MVA strain has been developed in the 1970s as a vaccine against smallpox but since the nineties it is widely tested in clinical trials as recombinant vector for vaccination or gene therapy applications. Biosafety aspects of MVA based viral vectors or vaccines have been recently reviewed [17,98].

Finally, several other novel viral-vectored sub-unit vaccines are under investigation, many based on simian adenoviruses. ChAdOx1 85A is a new candidate based on a simian (chimpanzee) adenoviral vector expressing the *Mtb* Ag 85A. ChAdOx1 is classified as a Human adenovirus serotype E. It differs from HuAd5 which belongs to serotype C [99]. The low prevalence of neutralizing antibodies against chimpanzee adenovirus in the human population makes it an excellent vaccine candidate. Furthermore, this vector has been used as a vaccine candidate against many pathogens and the results of pre-clinical and human clinical studies demonstrated its excellent safety and immunogenicity profile [100]. A phase I trial is ongoing to evaluate the safety of ChAdOx1 85A vaccination in healthy BCG-vaccinated subjects, with and without MVA-85A boost vaccination (NCT01829490). ChAdOx1 85A is administered by the intramuscular route. ChAdOx1 85A is made replication deficient by deletion of E1 and E3 genes [53]. The vectors are propagated in complementing cell line HEK 293.

Risk assessment of viral-based vectors is done case-by-case and should mainly consider the following points: (i) the intrinsic characteristics of the viral strain; (ii) the characteristics of the transgene; (iii) its biodistribution into the patient’s body and dissemination and (iv) the possibility of recombination with other viruses. Knowledge of the biodistribution of the viral vector is crucial to evaluate the risk associated with dissemination into the environment and possible transmission to people in close contact with the patient. General considerations on the biosafety of virus-derived vectors used in gene therapy and vaccination have been reviewed elsewhere [21].

#### 4.2.1. Intrinsic Characteristics of the Four Viral Strains Currently Used in TB Clinical Trials

In AERAS-402, the Ad35 has been made replication deficient by deletion of E1 genes which are necessary for expression of E2 and late genes required for adenoviral DNA synthesis, capsid protein expression, and viral replication. The E1 genes are deleted and replaced with an expression cassette with an exogenous promoter driving expression of the mycobacterial antigens (Ag85A, Ag85B and TB10.4). The vectors are propagated in complementing cell line PER.C6/55K which retains and expresses the E1A and E1B proteins [101].

Ad35 vectors can infect a variety of human cells because the primary receptor for Ad35, CD46, is ubiquitously expressed in human cells. Furthermore, Ad35 vectors efficiently transduce in the presence of anti-Ad5 antibodies.

As in AERAS-402, Ad5 is made replication deficient by deletion of E1 genes and by a subsequent deletion of the E3 genes. These genes are involved in anti-host immunity and are required for replication of the virus *in vitro*. The vectors are propagated in complementing cell line HEK 293 [52].

The majority of the transduced adenoviral vector genomes essentially remains episomal and integration into the host genome occurs only at very low frequency. Since adenoviral vectors do not integrate into the genome, dividing cells will gradually loose the adenoviral vector along with its transgene. While wild-type Ad belong to RG 2, the adenoviral vectors generally belong to the lower RG 1 as long as the genetic modifications lead to attenuation of the virus and the inserted gene(s) does not encode for potentially hazardous gene products.

MVA is a highly attenuated vaccinia strain derived from the Chorioallantoic Vaccine Ankara (CVA) strain belonging to the orthopox virus (OPV) genus. Serial passages in chicken embryo fibroblasts resulted in a genomic loss of approximately 15% compared to the parental CVA strain. This has reduced its virulence and pathogenesis making MVA unable to propagate in human and in most mammalian cells. It was found to be safe—safer than other vaccinia strains—and immunogenic. It has, as all poxviruses, a fully cytoplasmic cycle of propagation [102]. Hence the possibility of integration of genetic material from the virus in the host chromosomes is very low. In addition due to its high attenuation profile, risk of reconversion to wild-type is commonly accepted as negligible.

It is important to notice that most of the MVA strains are actually polyclonal and contain some variants capable to replicate in cells generally considered non-permissive for MVA. This has been observed *in vitro* but it has not been observed in any human clinical trial undertaken so far [103]. However information regarding the homogeneity of the MVA recipient strain is important in order to exclude the presence of replication competent MVA particles. Hence, the choice for a stable and homogenous MVA strain is recommended. Concerning MVA-85A used in clinical trials, no data on which precise MVA strain was used for its construction or regarding its homogeneity are publicly available.

In MVA-85A-IMX313 a small DNA sequence (IMX313) has been added in order to achieve an immune potentiating effect. It is an oligomerization domain which induces antigen multimerization and should result in increased immunogenicity without affecting Ag85A stability and expression [48].

As all poxviruses, MVA shows high environmental stability and high resistance to drying. However, if unintended environmental spreading occurs only limited environmental impact is expected due to the poor replicative and propagative characteristics of MVA [17]. In addition, vaccinia virus has no natural reservoir [104].

#### 4.2.2. The Characteristics of the Transgenes

The antigens 85A (Ag85A) and B (Ag85B) belong to a family of three highly homologous proteins: 85A, 85B and 85C. There are enzymes associated with mycolyl transferase activity and involved in the mycobacterial cell wall biosynthesis [105]. Ag85A is also involved in lipid storage body formation [106]. Ag85A, B and C are highly conserved in all mycobacterial species and Ag85A and Ag85B are major components of the *Mtb* cell wall and are immunodominant in *Mtb* infected animal and human studies. The expression of the gene encoding Ag85A or Ag85B in a recombinant viral vector vaccine is a way to induce and amplify the cellular immune response of the vaccine against *Mtb* [41].

Ag85A and Ag85B have no known toxic or allergic effects when administered to humans. To date MVA-85A has been administered to more than 1000 individuals with no vaccine related serious adverse effects [44]. It is not yet known if the presence of the multimerization domain IMX-313 as in MVA-85A-IMX313 changes the safety profile of the product. The first phase I trial with MVA-85A-IMX313 started in July 2013 (NCT01879163) and the results are not expected before 2015.

TB10.4 is a secreted protein encoded by the *Rv0288* gene. This gene was found to belong to a subfamily within the esat-6 family that consists of the three highly homologous proteins TB10.4, TB10.3 and TB12.9. The gene *Rv0288* is located in the esx cluster 3 that seems to be essential for the virulence of *Mtb* [107]. TB10.4 protein may play an important role in *Mtb* pathogenesis but until now its function is unknown [108]. This protein is recognized by human and murine T cells upon mycobacterial infection and is a target for antimycobacterial immune responses in humans. No information is available regarding potential toxicity of TB10.4 antigen. However AERAS-402 has been tested in clinical trials and no adverse effect has been observed in vaccinated healthy humans [49,50].

#### 4.2.3. Biodistribution and Dissemination of the Recombinant Viral Vector Vaccines

AERAS-402 and AdHu5Ag85A are administered by intra-muscular route. Biodistribution and shedding of Ad viral vectors are dependent on the dose and the route of administration and in case of intramuscular administration it depends on the muscle group that is injected [96,109]. Intramuscular administration of the Ad vector leads to systemic biodistribution and shedding via almost all excreta might occur. Shedding via semen and transport across the blood-brain barrier is not expected.

In pre-clinical studies AdHu5Ag85A and a bivalent Ad5 recombinant vector expressing the *Mtb* antigens 85A and TB10.4 have been administered by intranasal route in mice [52,110]. Intranasal route has not been tested in humans yet. Intranasal mucosal delivery of an Ad5 recombinant vector expressing green fluorescent protein to mice resulted in dissemination predominantly in the upper and lower respiratory tract. Dissemination to the olfactory bulb was moderate and only little or no viral dissemination to the brain has been observed [111].

It is concluded from an inventory of shedding data based on results reported for 201 patients that conversion to replication-competent virus has not been found in patients treated with a replication-deficient adenoviral vector [109]. In addition, the risk management could reduce significantly the likelihood of dissemination of the vector and infection of other persons.

The possible consequences of leakage of the vector into the environment are not known but may include, for example, adverse effects associated to the infection of personnel or people in general coming into contact with the vaccinated individuals or with contaminated surfaces or material, and recombination with other viruses.

If inadvertent horizontal transmission occurs with the replication-deficient adenoviral vectors considered here the risks would be minimal since the expressed proteins have no known harmful effect.

Infection of immunosuppressed persons with replication competent adenovirus (RCA) generated after recombination with a wild-type Ad (see below) could be responsible for adverse effects and specific measures should be taken to avoid contact with immunosuppressed persons two weeks after the injection of the vaccine.

MVA-85A tissue distribution studies in small animals were performed in pre-clinical studies together with toxicology tests [112]. Results are however not publicly available.

In a pre-clinical study performed with macaques where MVA-85A was administered via aerosol or intradermally the authors report that no viable virus was detected in any of the macaques tissue samples taken at necropsy 5 weeks after administration of MVA-85A [113]. Unfortunately no samples were taken earlier after the administration of the vaccine candidate.

Results of several phase I or II clinical trials with MVA-85A administered intradermally have been published: 13 in humans (infants, children, adolescents or adults) [34,35,36,37,38,39,40,41,42,43,44,46,47] and 2 in calves [114,115]. In none of these publication data are given about dispersion of the vector from the site of administration in the body of the treated subject (human or animal). Also no shedding assays are reported. Mild local skin reaction at the site of administration is frequently reported as adverse event in patients vaccinated with MVA-85A, and often scaling or desquamation is reported [34,35,38,39,42,43]. PCR assays on shed material complemented with *in vitro* cell culture on a permissive cell line could have been of great interest to assess if shedding of intact MVA vector occurs.

#### 4.2.4. Possibility of Recombination with Other Viruses

It cannot be excluded that the recombinant Ad exchanges its genetic material during co-infection of the same human cell by a wild-type adenovirus and thus reacquires a replication capacity generating RCA. The probability of occurrence of this event is extremely low because Ad vectors are non-replicative and would involve only a limited number of viral particles which would be rapidly eliminated by the immune system, and consequently would have no effects on health of the persons in contact with the vaccinated subject after a putative horizontal transmission. Individuals that have naturally been exposed to wild-type adenovirus develop neutralizing antibodies which constitute an additional barrier that protects against potential horizontal transmission of adenoviral vectors or RCA. These pre-existing neutralizing antibodies would likely not protect against adenoviral vectors derived from less common alternative adenoviral serotypes like Ad35.

For a recombinant MVA vaccine recombination with a naturally occurring homologous non human OPV is theoretically possible in case of co-infection and in this case some disrupted or deleted MVA genes could be rescued [116,117]. Such recombination could also result in the transfer of the transgene from the recombinant MVA to a replication competent OPV. It would require co-localization/co-infection of the same cells in the same host which is very unlikely, especially in humans, because there are no known human poxviruses. The likelihood increases if the vaccine is used for treatment of animals and if epidemiological data confirm the occurrence of natural OPV in the geographical area of vaccine administration. MVA-85A has been tested as vaccine for immunization of cattle against bovine tuberculosis [114,115]. This is a case where the question of potential recombination is certainly pertinent. However as the transgene has no known toxic effects the consequences of the transfer of Ag85A into a replication competent OPV are expected to be negligible. In addition, due to its high attenuation profile, the risk of the recombinant MVA to re-acquire the wild-type characters is commonly accepted as negligible [118,119].

#### 4.2.5. Risk Classification

Human Adenoviruses belong to RG 2 but when made replication deficient by deletion of the E1A and E1B regions, which is the case for AERAS-402 and AdHu5Ag85A, they could be classified into RG 1 for which level 1 containment is appropriate. MVA belongs to the same risk group and the same biosafety profile of MVA has been observed upon administration to immune-compromised macaques [120].

The final risk classification of recombinant viral vector vaccines has to take into account any potential risk associated with the transgene. Based on their biological properties, there are no reasons to think that the presence of Ag85A in the four vaccines under consideration here (AERAS-402, AdHu5Ag85A, MVA-85A and MVA-85A-IMX313) or the presence of Ag85B and TB10.4 in AERAS-402 could change the safety profile of the recombinant viruses. As previously mentioned, the three antigens Ag85A, Ag85B and TB10.4 have no known toxic or allergic effects when expressed in humans [49,50,51]. However it is important to investigate whether the insertion of the gene of interest has altered the virological safety profile of the recipient viral strains. The results obtained in the clinical trials with AERAS-402 and AdHu5Ag85A have shown that these vaccines are safe and no adverse effects have been observed in phase I clinical trials [49,50,51,101]. The results obtained in the clinical trials with MVA-85A are reassuring: to date MVA-85A has been administered to more than 1000 individuals, including infants [38,45,46], HIV infected persons [39,40,44], patients previously vaccinated with BCG [34,35,36,38,41,43,47] and patients latently infected with *Mtb* [37,39], with no vaccine-related serious adverse event. In all these clinical trials but one the vaccine was administered intradermally. Only in one trial MVA-85A was also administered via the intramuscular route [47]. Due to the properties of the transgenes and because MVA and Ad are defective for the replication, the viral vectors MVA-85A, AERAS-402 and AdHu5Ag85A can be classified into RG 1. Regarding MVA-85A-IMX313 more pre-clinical and clinical data are needed before assigning this viral vector vaccine into a risk group. It should be investigated if the presence of the IMX313 adjuvant could potentially result in an exacerbated immune response.

#### 4.2.6. Environmental Risk Assessment

Only a limited environmental impact is to be expected after unintended environmental spreading of MVA based vaccines, like both MVA-based TB vaccines under consideration here, due to their poor replicative and propagative characteristics (see above). In addition, if an external person is exposed no toxic effect is expected from the new introduced gene. The same applies to the recombinant Ad5 and Ad35. In addition as human Ads are mostly species specific Ad5 and Ad35 are not pathogenic to animals.

#### 4.2.7. Risk Management Measures

Regarding MVA based vaccines it is known that MVA strains have variants which may replicate in human cells. Therefore during the production of MVA TB vaccines the absence of replicating viruses should be attested by infection assays [121].

The attenuation in AERAS-402, AdHu5Ag85A, MVA-85A and the history of safe use of MVA allows handling these recombinant viruses under CL-1 in the clinical setting. As mentioned before the addition of the Ag85 gene(s) has not changed their safety profile. During the production process, all batches should be tested for the presence of RCA.

For personnel manipulating AERAS-402, AdHu5Ag85A, MVA-85A and MVA85A-IMX13, the primary hazards consist in exposure to droplets or aerosols of mucous membrane or broken skin, and inadvertent parenteral inoculation (injury with needle stick or other sharp objects). There is also a risk of accidental projection of the vaccine candidates into the eye or other mucous membranes, or unintentional contamination via close contact with contaminated material. In addition, such bio-incidents could result in unintentional dissemination of the recombinant viruses in the environment. Exposure via these pathways can be prevented by application of risk management strategies. Aerosol producing operations should be reduced during preparation and administration of the viral vectors and personnel manipulating the vectors should wear adequate protective clothing such as lab coats, gloves, goggles and masks because the puncture of a container holding the vector may produce aerosols. Some manipulations should preferably be carried out in a class II biosafety cabinet. Work with needles and other sharp objects should be strictly limited and workers should never recap nor remove needles from syringes.

As MVA and Ads are relatively stable, spills should be inactivated by an appropriate disinfectant, allowing sufficient contact time before disposal. Contaminated waste and personal protective equipment should be inactivated using an appropriate method before disposal and potentially contaminated non-disposable materials need to be properly decontaminated. If an incident occurs that could lead to infection (e.g., breakage of a vial containing the vector, or needle piercing), applicable first aid should be performed (*i.e.*, flushing eyes for ocular exposure, placing an absorbent tissue on the affected area in order to absorb all viral particles and apply disinfectant directly to the tissue for percutaneous exposure), followed by reporting to the supervisor.

Special precautions against aerosol exposure of care givers should be taken if recombinant TB vaccine is delivered by the aerosol route. This route of administration has not yet been tested in clinical trials but aerosol delivery of MVA-85A was tested in mice and macaques. The aerosol route could potentially circumvent immunity against MVA induced by previous vaccinations [113]. In mice aerosol vaccination strongly increases the Ag85A specific CD4^+^ T cell response in the draining lung lymph nodes [122] and in macaques aerosol vaccination by direct delivery to the lungs prevented anti-vector antibody response [113]. In addition the triggering of immune response in the mucosa-associated lymphoid tissue might lead to increased protection against pulmonary TB. Inducing the immune response at the site where the pathogen is first encountered would allow a faster local response [113]. If for efficacy or economic reasons the aerosol route should be chosen for the vaccination of humans it changes the exposure pathway through which the MVA-85A may interact with personnel, patient relatives and/or the environment. Such a change should be taken into account in the risk assessment.

Personnel manipulating recombinant TB vaccines could latently be infected with *Mtb*. Also the subject treated with the recombinant vaccine could possibly come into contact with caregivers or relatives who are immunosuppressed, HIV infected, latently infected with *Mtb* or previously vaccinated with BCG. Are extra precautions needed to prevent any advertent contact of these persons with the recombinant virally vectored TB vaccines? In clinical trials where MVA-85A has been administered to HIV infected subjects [39,40,44], to healthy subjects latently infected with *Mtb* [37,39] or to subjects previously vaccinated with BCG [34,36,38] no serious adverse events were observed and in particular no Koch reaction has been observed. This indicates that MVA-85A is not a threat for HIV infected people, persons latently infected with *Mtb* or previously vaccinated with BCG.

Ad infection can be dangerous in immunosuppressed individuals. Although the risk is certainly much lower with replication defective Ads, after the injection of a recombinant Ad vaccine, the patient should avoid as much as possible contacts with immunosuppressed persons and naïve infants or children during 2 weeks. There are no drugs approved to treat specifically Ad infections. Nevertheless some antiviral drugs are used in the clinic [95].

## 5. Conclusions and Perspectives

Success of TB vaccines currently evaluated in clinical trials is unpredictable and in this respect, 2013 has been disappointing. Evidence for efficacy of most of the vaccine candidates is poor compared to BCG vaccine, including in animal models of pre-clinical studies. In March 2013 the results of the MVA-85A-AERAS-485 phase IIb clinical trial (NCT00953927) were published [46]. In this trial, which was conducted in South Africa, healthy infants (aged 4–6 months) that were previously vaccinated with BCG received one intradermal dose of MVA-85A or an equal volume of Candida skin test antigen as placebo. Though the primary objective of this trial was safety, also efficacy evaluation against TB disease (diagnosed by microbiological, radiological and clinical criteria) or *Mtb* infection (defined by QuantiFERON TB Gold In-tube conversion) was performed in a defined group of participants. Safety results were satisfactory, while no improved efficacy was observed in the group of infants that were BCG-primed and MVA-85A-boosted *vs.* BCG-primed and placebo-boosted infants.

In retrospect, a number of factors can explain lack of improved efficacy of this vaccination scheme [123]. In our opinion, the main point is related to the population that was evaluated in this study, namely infants that had been BCG vaccinated early after birth. Indeed, in this population, BCG is performing well in terms of protective efficacy against childhood forms of TB. In addition, MVA-85A proved modestly immunogenic when levels of vaccine-induced cellular immunity were compared to the levels achieved when BCG-vaccinated adults are vaccinated with MVA-85A as a booster vaccine. Hence, the efficacy results obtained in this trial do not exclude the possibility that a boost with MVA-85A administered to adults that have been vaccinated with BCG during infancy or that are latently infected with *Mtb* might prove efficient in terms of reduction of TB morbidity in ongoing and future clinical trials. Concerning the modest immunogenicity observed in infants boosted with MVA-85A, it is worth mentioning that a number of clinical trials are ongoing to improve this aspect. In these trials, MVA-85A has been either modified by fusing Ag85A to the oligomerization domain IMX313 (NCT01879163) or vaccination protocols combining MVA-85A with other viral-vectored sub-unit vaccines are being tested. Such being the case in clinical trial NCT01683773 in which booster immunization with AERAS-402 (Crucell Ad35) is followed by a second booster vaccination with MVA-85A, or clinical trial NCT01829490 in which ChAdOx1 85A (based on a chimpazee adenoviral vector expressing Ag85A) is followed by a second booster vaccination with MVA-85A.

In addition, it is important to emphasize that NCT00953927 was the first phase IIb trial for a TB vaccine conducted in a high-burden setting and the solutions, which were implemented for this trial will be of value in the planning of efficacy trials of other TB vaccine candidates [45].

Nevertheless, as success of the TB vaccines currently evaluated in clinical trials is unpredictable, it is also essential to continue fundamental and pre-clinical research to better understand protective immunity to *Mtb* and to identify novel TB vaccine candidates that might prove more efficient than the candidates currently under clinical evaluation.

Promising novel “BCG replacement” candidates are either based on genetic modification of BCG or on genetic attenuation of virulent *Mtb*. Some examples of interesting recombinant BCG candidates are the genetically modified BCG *∆zmp1* [62] and BCG *∆sapM* [61]. Both deletions involve genes that negatively regulate the process of phagosome maturation. Hence their deletion should favor antigen escape from the phagosomal compartment to increase cross-presentation of mycobacterial antigen over those achieved by parenteral BCG. Indeed, the zinc metalloprotease 1 (Zmp1) is required to arrest phagosome maturation and SapM is a secreted acid phosphatase, which plays a critical role during phagosome maturation.

Concerning candidates based on attenuation of *Mtb*, strains lacking the *SapM* gene have been described as well as pantothenate auxotrophs strains [124,125]. In addition, two highly attenuated *Mtb* mutants, MGM1991 and *Mtb∆hma::hyg* (HMA) have recently been proposed as potential vaccine candidates against TB [126]. These strains lack all oxygenated mycolates in their cell wall, which are *Mtb* glycolipids that have been associated with *Mtb* pathogenesis in mice, in part through effects on the inflammatory activity of trehalose dimycolate (cord factor). As compared to vaccination with BCG, the subcutaneous vaccination of C57BL/6 mice with either of the two mutants induced stronger Th1 (IFN-γ, IL-2 and TNF-α) and IL-17 responses. In addition, compared with BCG vaccination, significantly higher number of mycobacteria-specific IFN-γ producing CD4^+^ and CD8^+^ T cells were detected and stronger protection against *Mtb* challenge was observed in mice vaccinated with the *Mtb* mutants. For the *Mtb*-based vaccine candidates the risk assessment procedure will need to thoroughly assess any virulence and possible virulence reversion, as it has been described for MTBVAC vaccine candidate.

Besides safety and efficacy of the novel vaccine candidates against TB, biosafety should also be considered when this vaccine is composed of a GMO. Biosafety focuses on the protection of public health and the environment from an intended or unintended exposure to the GMO-based vaccine by implementing adapted containment measures during handling of the GMO. The risk assessment of future clinical trials with new GMO-based vaccine candidates will follow the same procedure of risk analysis as that followed for current clinical trials using GMO-based vaccines: what are the intrinsic characteristics of the parental micro-organism (or recipient organism), what is the function of the transgene or the mutated gene in the GMO and what are the characteristics of the final GMO? In case of an accidental release into the environment, what could be the consequences of an exposure of the environment, the human being or of animals to this GMO? Identification of potential risks of handling the GMO vaccine and the probability of their occurrence should define the risk management of the clinical trial. It has to be noted that at each step of the development of a GMO vaccine the risk assessment should take into account any new data generated by the previous steps.

Concerning the risk assessment procedure for new BCG- and *Mtb*-based candidates, it is important to note that these recombinant vaccines have been designed by targeted inactivation of endogenous genes and not by expression of heterologous virulence genes (as it has been the case for VPM1002 or for AERAS-422). Hence, the risk assessment procedure will need to take into account the genetic stability of the vaccine candidates. Particular attention is required with live vaccines based on attenuated pathogens on their capacity of virulence reversion as well as on their capacity to multiply and propagate in the vaccinated host and in the environment.

The successful use of viral vector vaccines against TB has not been demonstrated so far. However research is still ongoing and this review has discussed the biosafety issues which need to be considered carefully when performing clinical trials with recombinant Adenovirus or MVA. Although a case-by-case risk assessment should be performed on each clinical trial involving GMO-based vaccines, the same general methodology can be applied.
